# Preliminary Results of an Ongoing Prospective Clinical Trial on the Use of ^68^Ga-PSMA and ^68^Ga-DOTA-RM2 PET/MRI in Staging of High-Risk Prostate Cancer Patients

**DOI:** 10.3390/diagnostics11112068

**Published:** 2021-11-09

**Authors:** Paola Mapelli, Samuele Ghezzo, Ana Maria Samanes Gajate, Erik Preza, Giorgio Brembilla, Vito Cucchiara, Naghia Ahmed, Carolina Bezzi, Luca Presotto, Valentino Bettinardi, Annarita Savi, Patrizia Magnani, Raffaele Menichini, Angela Coliva, Ilaria Neri, Ettore Di Gaeta, Luigi Gianolli, Massimo Freschi, Alberto Briganti, Francesco De Cobelli, Paola Scifo, Maria Picchio

**Affiliations:** 1 Vita-Salute San Raffaele University, 20132 Milan, Italy; mapelli.paola@hsr.it (P.M.); ghezzo.samuele@hsr.it (S.G.); brembilla.giorgio@hsr.it (G.B.); cucchiara.vito@hsr.it (V.C.); bezzi.carolina@hsr.it (C.B.); briganti.alberto@hsr.it (A.B.); decobelli.francesco@hsr.it (F.D.C.); 2 Department of Nuclear Medicine, IRCCS San Raffaele Scientific Institute, 20132 Milan, Italy; samanesgajate.anamaria@hsr.it (A.M.S.G.); preza.erik@hsr.it (E.P.); presotto.luca@hsr.it (L.P.); bettinardi.valentino@hsr.it (V.B.); savi.annarita@hsr.it (A.S.); magnani.patrizia@hsr.it (P.M.); menichini.raffaele@hsr.it (R.M.); coliva.angela@hsr.it (A.C.); neri.ilaria@hsr.it (I.N.); gianolli.luigi@hsr.it (L.G.); scifo.paola@hsr.it (P.S.); 3 Department of Radiology, IRCCS San Raffaele Scientific Institute, Via Olgettina 60, 20132 Milan, Italy; digaeta.ettore@hsr.it; 4 Department of Urology, Division of Experimental Oncology, URI, Urological Research Institute, IRCCS San Raffaele Scientific Institute, 20132 Milan, Italy; 5 Department of Pathology, IRCCS San Raffaele Scientific Institute, Via Olgettina 60, 20132 Milan, Italy; ahmed.naghia@hsr.it (N.A.); freschi.massimo@hsr.it (M.F.)

**Keywords:** hybrid imaging, PET/MRI, PSMA, RM2, prostate cancer, multimodal imaging

## Abstract

The aim of the present study is to investigate the synergic role of ^68^Ga-PSMA PET/MRI and ^68^Ga-DOTA-RM2 PET/MRI in prostate cancer (PCa) staging. We present pilot data on twenty-two patients with biopsy-proven PCa that underwent ^68^Ga-PSMA PET/MRI for staging purposes, with 19/22 also undergoing ^68^Gaa-DOTA-RM2 PET/MRI. TNM classification based on image findings was performed and quantitative imaging parameters were collected for each scan. Furthermore, twelve patients underwent radical prostatectomy with the availability of histological data that were used as the gold standard to validate intraprostatic findings. A DICE score between regions of interest manually segmented on the primary tumour on ^68^Ga-PSMA PET, ^68^Ga-DOTA-RM2 PET and on T2 MRI was computed. All imaging modalities detected the primary PCa in 18/19 patients, with ^68^Ga-DOTA-RM2 PET not detecting any lesion in 1/19 patients. In the remaining patients, ^68^Ga-PSMA and MRI were concordant. Seven patients presented seminal vesicles involvement on MRI, with two of these being also detected by ^68^Ga-PSMA, and ^68^Ga-DOTA-RM2 PET being negative. Regarding extraprostatic disease, ^68^Ga-PSMA PET, ^68^Ga-DOTA-RM2 PET and MRI resulted positive in seven, four and five patients at lymph-nodal level, respectively, and at a bone level in three, zero and one patients, respectively. These preliminary results suggest the potential complementary role of ^68^Ga-PSMA PET, ^68^Ga-DOTA-RM2 PET and MRI in PCa characterization during the staging phase.

## 1. Introduction

Prostate cancer (PCa) is one of the worldwide leading causes of cancer-related death. Approximately 15% of men present with high-risk PCa, which is characterized by an increased risk of extracapsular extension, locally advanced disease, and/or bone metastases [1]. Hence, at diagnosis, a whole-body staging for high-risk PCa patients is strongly recommended regardless of the surgical or radiation-based treatment decision [2].

The current staging of intermediate and high-risk PCa includes imaging of abdomen and pelvis performed by using Computed Tomography (CT) or Magnetic Resonance Imaging (MRI) and bone scan to evaluate potential sites of metastatic spread.

The current EAU-ESTRO-SIOG guidelines report that Positron Emission Tomography/CT (PET/CT) is a valuable imaging modality that might be considered in men with high-risk diseases undergoing initial staging [3]. However, as no randomised-control trials demonstrating survival benefit are available yet, its role in guiding therapeutic decisions must be cautious [3].

Multi-parametric MRI (mp-MRI) is a well-established imaging modality for PCa assessment and it is used to detect the primary tumour, guide biopsies and define the local extent of the disease; its usefulness for local staging has been largely reported, although local staging with MRI might be associated with limited sensitivity [4,5,6].

Molecular imaging with PET represents a valid imaging approach in PCa staging, with new PET tracers other than Choline having a relevant role in improving diagnoses, staging and follow-up of PCa [7,8,9,10].

In this regard, prostate-specific membrane antigen (PSMA), a transmembrane protein with a significantly increased expression in PCa cells, is an imaging probe that has been introduced in clinical practice, with recent data demonstrating good accuracy in PCa staging [11,12].

Gastrin releasing peptide receptor (GRPR) is a G-protein coupled receptor overexpressed in different types of cancer including PCa [13,14]. The ^68^Ga-DOTA-RM2 is a GRPR antagonist used as a PET imaging probe that has demonstrated promising, but still limited results in PCa imaging [15,16,17].

Hybrid PET/MRI allows for the simultaneous acquisition of metabolic, structural, and functional imaging information regarding PCa status in a whole-body single session examination, thus representing an innovative imaging approach capable to overcome the pitfalls of conventional imaging and, potentially, helping clinicians in the management of PCa. Only a few, preliminary studies have compared ^68^Ga-PSMA and ^68^Ga-DOTA-RM2-PET radiotracers in PCa by using PET/CT or PET/MRI so far, with promising results in both patients presenting with biochemical recurrence and in those with newly diagnosed intermediate- or high-risk prostate cancer [15,18,19].

The aim of the present study is to report our preliminary experience on the synergic use of ^68^Ga-PSMA PET/MRI and ^68^Ga-RM2 PET/MRI in prostate cancer staging.

## 2. Materials and Methods

### 2.1. Patients

In this prospective clinical study, 22 patients with biopsy-proven PCa were enrolled from 1 September 2020 to 31 August 2021 at the IRCCS San Raffaele Scientific Institute.

Inclusion criteria were age greater than 18 years at the time of PET/MRI scan, biopsy-proven high-risk PCa (defined as PSA > 20 ng/mL and/or clinical stage ≥ cT2c and/or biopsy ISUP grade ≥ 4, according to European Association of Urology guidelines [3]) candidate to prostatectomy and pelvic lymphadenectomy. Exclusion criteria were inability to complete the required imaging examinations (i.e., severe claustrophobia), medical condition possibly interfering and significantly affecting study compliance, all contraindications to undergo MRI scan (i.e., metallic/conductive or electrically/magnetically active implants without MR-safe or MR-conditional labelling) and evidence of metastatic disease on conventional imaging contraindicating the surgical procedure.

All recruited patients underwent ^68^Ga-PSMA PET/MRI, with 19 also undergoing ^68^Ga-DOTA-RM2 PET/MRI in two different days, with at least 48h interval, for staging purposes before radical prostatectomy. Histological validation of imaging findings was retrieved from clinical reports for all patients who have undergone radical prostatectomy, so far.

This study was approved by the Institutional Ethics Committee of IRCCS San Raffaele Scientific Institute (EudraCT: 2018-001034-18) and all patients gave written informed consent to participate in the study.

### 2.2. ^68^Ga-PSMA PET/MRI Acquisition Protocol

^68^Ga-PSMA-11 was synthesised by a fully automated synthesis module (Neptis Mo-saic-RS, ORA, Neuville, Belgium) connected to a ^68^Ge/^68^Ga generator (1.85 GBq Galli Ad, IRE ELiT, Fleurus, Belgium) and equipped with a disposable single-use cassette kit (ABX GmbH, Radeberg, Germany). A standardised labelling sequence with 15 μg (15 nmol) of unlabelled PSMA 11 (ABX GmbH) was used. The final product was sterilely filtered over 0.22 μm PVDF filters. For quality control, ^68^Ga-PSMA 11 was analysed by radio analytic high-performance liquid chromatography on a modular system (Waters) equipped with a diode array detector and a radio detector using an RP-18 column (ACE 5 μm C18, 150 × 3 mm, Advanced Chromatography Technologies Ltd., Aberdeen, Scotland). A gradient elution over 13 min at a flow of 1.5 mL/min from 90%A to 30%A and again 90%A was employed, where Solvent A was Water + 0.1% TFA and Solvent B was CH3CN+ 0.1% TFA. Other quality controls performed before release included TLC on iTLC strips with MeOH/1M AcONH4, pH measurement and radionuclidic purity. Residual HEPES determination, Ethanol quantification and Microbiological purity were assessed on decayed product. Uncorrected radiochemical yield was over 70% with a radiochemical purity > 91%.

Fasting condition was requested on the day of ^68^Ga-PSMA PET/MRI scan. Images were acquired on a SiPM-based TOF-PET GE Signa PET/MRI 3 Tesla system (GE Healthcare, Waukesha, WI, USA) from the skull base to mid-thigh. The ^68^Ga-PSMA PET/MRI scan started approximately 60 min (mean ± SD, 63 ± 9 min) after injection of 122–255 MBq (mean ± SD, 170 ± 33 MBq) of ^68^Ga-PSMA.

The ^68G^a-PSMA PET/MR examination protocol included a high statistic (HS) scan (20 min), covering a single bed position, that was simultaneously acquired to the following MR sequences:

- an axial T2 weighted sequence with large field of view (FOV): FSE, TR = 10235 ms; TE = 99.7 ms, FOV = 32 × 32 cm^2^; voxel size = 0.9 × 0.9 × 5 mm^3^;
- an axial T2 weighted sequence with small FOV: PROPELLER, TR = 9578 ms, TE = 151 ms, FOV = 18 × 18 cm^2^, voxel size = 0.6 × 0.6 × 3 mm^3^,
- a coronal T2 weighted sequence with small FOV: PROPELLER, TR = 9578 ms, TE = 151 ms, FOV = 18 × 18 cm^2^, voxel size= 0.6 × 0.6 × 3 mm^3^,
- a diffusion weighted imaging (DWI) sequence with small FOV: TR = 6643 ms, TR = 79.5 ms, FOV = 18 × 9 cm^2^, voxel size = 1.8 × 1.8 × 3 mm^3^; b = 50, 800, 1400 s/mm^2^
- T1-Lava Flex sequence of the whole pelvic region pre-contrast and post-contrast: TR = 5 ms, TE = 1.7 ms, FOV: 44 × 35.2 cm^2^, voxel size = 1.3 × 1.2 × 2 mm^3^
- a high temporal resolution T1 perfusion sequence after IV injection of 0.1 mmol/kg bolus of gadobutrol (Gadovist, Bayer Schering Pharma, Germany) at a flow rate of 3.5 mL/s: DISCO, TR = 5.1 ms, TE = 1.7 ms, FOV = 29 × 29 cm^2^, Voxel size = 1.9 × 2.2 × 3 mm^3^, 88 dynamics.

Following the single bed acquisition, a total-body (TB) PET scan (5–6 FOVs, 4 min/FOV) was then simultaneously acquired to an MRI TB T1 Lava Flex sequence and a TB DWI with b = 50, b = 1000 s/mm^2^.

PET images were reconstructed using a Bayesian penalised likelihood reconstruction algorithm [20] with a reconstructed FOV of 60 cm and image matrix of 192 × 192. The algorithm includes a Point Spread Function and Time of Flight information.

Attenuation Correction (AC) of PET data was performed using MR AC technique based on the processing of the LAVA-Flex sequences acquired simultaneously with the PET data.

### 2.3. The ^68^Ga-DOTA-RM2 PET/MRI Acquisition Protocol

The ^68^Ga-DOTA-RM2 was synthesised by a kit-like procedure developed for the radio-labelling with GalliAd^®^ generator (IRE Elite, Fleurus, Belgium). Briefly, the eluate from the ^68^Ge/^68^Ga generator (1.85 GBq Galli Ad, IRE ELiT, Fleurus, Belgium) was added to a sterile vial containing 40 µg of DOTA-RM2 (Life Molecular Imaging, Fribourg, Germany) in format buffer and ascorbic acid. Reaction vial is placed in a pre-heated thermostat at 115 °C for 10 min. Successively, vial is left to cool down for 10 min at room temperature. No purification step was needed. The solution is sterile filtered over sterile 0.22 μm PVDF membrane and dispensed as injectable solution. For quality control, ^68^Ga-DOTA-RM2 was analysed by radio analytic high-performance liquid chromatography on a modular system (Waters) equipped with a diode array detector and a radio detector using an RP-18 column (ACE 5 μm C18, 150 × 3 mm, Advanced Chromatography Technologies Ltd., Aberdeen, Scotland). A gradient elution over 16 min at a flow of 1.0 mL/min from 80%A to 20%A and again 80%A was employed, where Solvent A was Water + 0.1% TFA and Solvent B was CH3CN + 0.1% TFA. Unbound gallium was quantified by iTLC strips with MeOH/1M AcONH4 while ionic gallium with TLC strips with 0.1 M sodium citrate pH5. pH of the final solution was 3.2–3.8. Radionuclidic purity was assessed before release, microbiological purity was assessed on decayed product. Uncorrected radiochemical yield was over 60% with a radio-chemical purity > 91%.

As for ^68^Ga-PSMA PET/MRI, fasting condition was requested on the day of the examination and the same PET/MRI scanner was used.

Images were acquired from the base of the skull to mid-thigh and started approximatively 50 min (mean ± SD, 54 ± 8 min) after injection of 74–222 MBq (mean ± SD, 164 ± 33 MBq) of ^68^Ga-DOTA-RM2.

The ^68^Ga-DOTA-RM2 PET/MR examination protocol included an HS scan (20 min), covering a single bed position, that was simultaneously acquired to the following MR sequences: an axial T2 weighted sequence with large FOV (32 × 32 cm^2^), an axial 3D T2 sequence with small FOV, a T1-Lava Flex sequence of the whole pelvic region. Following the single bed acquisition, a TB PET scan (5–6 FOVs, 4 min/FOV) was then simultaneously acquired with a TB axial Lava Flex sequence and a TB sagittal STIR sequence on the spine. Reconstruction and attenuation correction of PET images were performed by using the same algorithms and parameters used for ^68^Ga-PSMA PET images.

### 2.4. PET/MR Image Analysis

A ^68^GA-PSMA and ^68^Ga-DOTA-RM2 image read-out was performed on the Advantage Workstation (AW, General Electric Healthcare, Waukesha, WI, USA) on which PET, MRI and fused PET/MRI images could be visualized in axial, coronal and sagittal planes. HS PET acquisition bed on the pelvic region and TB PET examination of both ^68^Ga-PSMA and ^68^Ga-DOTA-RM2 PET images were qualitatively interpreted by two experienced (more than 10 years of experience) Nuclear Medicine physicians, with knowledge of all the available patients’ clinical and imaging information.

For primary tumour assessment, HS and TB pelvic PET images were qualitatively evaluated for both ^68^Ga-PSMA and ^68^Ga-DOTA-RM2. The presence of ^68^Ga-PSMA and ^68G^a-DOTA-RM2 increased uptake was considered positive for malignancy with the anatomical site being defined on the basis of MRI anatomy, except for those areas of physiologically increased uptake [21,22]. Regions of interest (ROIs) on the primary tumour, showing ^68^Ga-PSMA and ^68^Ga-DOTA-RM2 uptake on HS PET images were semi-automatically defined on transaxial PET images. Furthermore, the following semi-quantitative parameters were calculated for the primary tumour on HS PET images for both radiotracers: maximum standardised uptake value (SUVmax), mean SUV (SUVmean, using different thresholds, namely 40%, 50%, 60% of the maximum value-SUVmean40, SUVmean50, SUVmean60) and metabolic tumour volume (MTV) calculated at different thresholds (MTV40, MTV50, MTV60).

In addition, to determine the volume and the location of ^68^Ga-PSMA and ^68^Ga-DOTA-RM2 PET primary tumour uptake, one experienced Nuclear Medicine physician manually segmented the primary tumour slice-by-slice using 3D Slicer software (revision 29402) [23] on both ^68^Ga-PSMA and ^68^Ga-DOTA-RM2 PET images. Afterward, all segmentations were co-registered and brought to a common reference volume (one of the first PET studies). To do that, the MRAC of the ^68^Ga-DOTA-RM2 PET study was firstly co-registered to the one of the ^68^Ga-PSMA PET, by means of 3D Slicer, and then the obtained transformations were applied to the ^68^Ga-DOTA-RM2 PET images and the corresponding ROI segmentation.

After the evaluation of the primary prostatic tumour, the whole-body distribution pattern of both ^68^Ga-PSMA and ^68^Ga-DOTA-RM2 were qualitatively assessed, and the presence of extra-prostatic ^68^Ga-PSMA and ^68^Ga-DOTA-RM2 increased uptake was considered positive for malignancy, with the exception of areas of physiologically increased uptake. The number and the site of lymph nodal involvement were reported, as well as the presence of suspect distant metastases. The anatomical site was defined on the basis of MRI anatomy.

In case of suspect bone metastasis in PET images, the whole-body MRI sequences were screened to confirm the spreading of the disease.

MR images acquired during HS ^68^Ga-PSMA PET were initially processed using AW software: small FOV DWI with b values of 50–800 were used to generate ADC maps. Volumetric ROIs of lesions visible to T2 and ADC images were created using 3D Slicer to obtain the following quantitative parameters: lesion volume, mean ADC (ADCmean) and minimum (ADCmin).

### 2.5. Qualitative and Quantitative Comparison of ^68^Ga-PSMA, ^68^Ga-DOTA-RM2 and MRI 

A qualitative comparison between ^68^Ga-PSMA and ^68^Ga-DOTA-RM2 intra-prostatic uptake and morphological findings detected on MR images was performed in order to describe the possible concordances and discrepancies between metabolic and morphologic imaging. Moreover, a qualitative comparison was also performed in terms of number and sites of lymph nodal and distant metastases for all patients, considering all three different imaging modalities.

Finally, DICE score between the ROIs manually segmented on the primary tumour on ^68^Ga-PSMA PET and ^68^Ga-DOTA-RM2 PET and on MRI was computed in order to evaluate the correspondence of the intra-prostatic findings referable to the site of primary tumour across modalities.

### 2.6. Correlations between PET Semi-Quantitative and MRI Quantitative Imaging Parameters

To provide improved characterization of the primary tumour, a correlation between multitracer PET and MRI parameters was performed. In particular, a Spearman correlation was calculated between the semi-quantitative PET parameters measured on HS ^68^Ga-PSMA PET and HS ^68^Ga-DOTA-RM2 PET images (SUVmax, SUVmean40, SUVmean50, SUVmean60, MTV40, MTV50, MTV60, manually segmented lesion volume), the quantitative parameters measured on MRI (manually segmented lesion volume, ADCmin, ADCmean) and clinical data (PSA, Gleason Score, ISUP score). A *p*-value < 0.05 was considered statistically significant.

## 3. Results

### 3.1. Patients

Twenty-two men (mean age: 65 years; range 52–80) with biopsy-proven high-risk PCa were enrolled in this prospective pilot study, so far. All patients had a Gleason score ≥ 7 on biopsy, with a mean PSA at time of diagnosis of 7.40 ng/mL (range: 2.5–26.17). Patients’ characteristics are reported in Table 1. All patients underwent ^68^Ga-PSMA PET/MRI and 19/22 also underwent ^68^Ga-DOTA-RM2 PET/MRI within sixteen days (mean: 3 days, range: 2–16 days) because of reduced compliance to the study protocol. Twelve patients have undergone radical prostatectomy so far, with the availability of histological data that were used as a reference standard to validate imaging findings.

### 3.2. PET/MRI Findings

**Figure 1:**
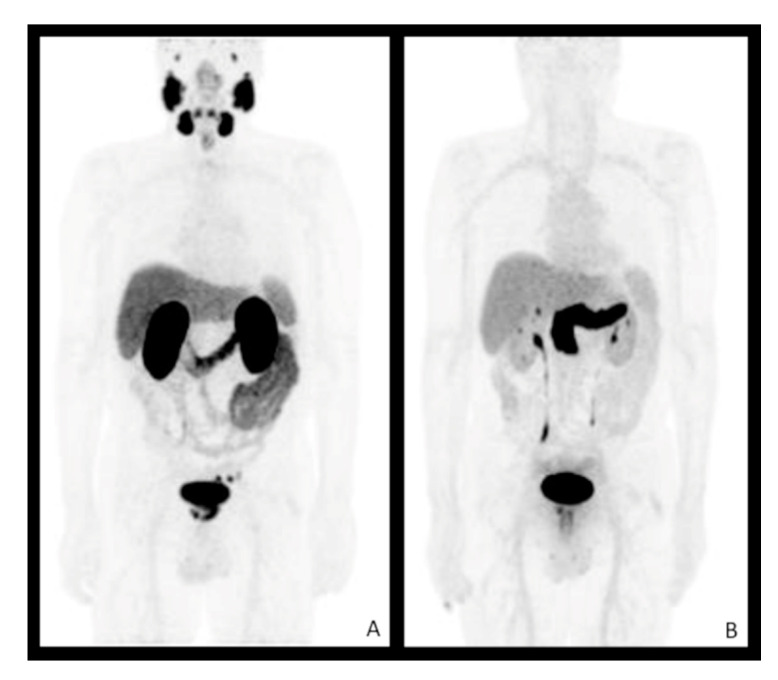
Physiological biodistribution of ^68^Ga-PSMA (**A**) and ^68^Ga-DOTA-RM2 (**B**) in patient n. 3.

An example of whole-body biodistribution of ^68^Ga-PSMA PET and ^68^Ga-DOTA-RM2 PET is reported in Figure 1. Physiological high ^68^Ga-PSMA uptake can be visualised in the salivary and lacrimal glands, liver, spleen, small intestine, kidneys, urinary bladder and ureters (Figure 1A), while ^68^Ga-DOTA-RM2 showed physiological high uptake in the pancreatic gland and urinary bladder (Figure 1B).

^68^Ga-PSMA PET detected intra-prostatic lesions in all patients, while ^68^Ga-DOTA-RM2 PET identified the intraprostatic disease in 18/19 patients. Additionally, in 2/22 patients ^68^Ga-PSMA PET also detected seminal vesicles uptake. The specific sites of intra-prostatic ^68^Ga-PSMA and ^68^Ga-DOTA-RM2 uptake are reported in Table 2.

The analysis of semi-quantitative parameters of prostate uptake extracted from HS ^68^Ga-PSMA images showed a mean SUVmax of 26.16 (range: 4.08–73.92), SUVmean40–50–60% of 15.79, 17.62 and 19.61, respectively (ranges: 3.02–43.85; 3.04–50.37; 3.18–54.61, respectively), MTV40–50–60% of 2.55, 1.75 and 1.13, respectively (ranges: 0.19–15.53; 0.14–9.77 and 0.08–5.97, respectively). All ^68^Ga-PSMA PET-derived parameters, obtained from HS PET images simultaneously acquired with dedicated MRI acquisition on the pelvis, are reported in Table 3.

Similarly, semi-quantitative parameters derived from HS ^68^Ga-DOTA-RM2 PET images of prostate uptake showed a mean SUVmax of 15.40 (range: 3.39–30.93), SUVmean40–50–60% of 9.86, 10.90and 11.69, respectively (ranges: 2.88–20.33; 2.99–22.62; 3.09–23.55, respectively), MTV40–50–60% of 3.13, 2.37 and 1.88, respectively (ranges: 0.60–7.09; 0.35–5.62 and 0.24–4.70, respectively). All ^68^Ga-DOTA-RM2 PET-derived parameters, obtained from HS PET images simultaneously acquired with dedicated MRI acquisition on the pelvis, are reported in Table 4.

TB ^68^Ga-PSMA images revealed a suspicion for lymph nodal involvement in 7/22 patients, and for bone involvement in 3/22. In TB ^68^Ga-DOTA-RM2 PET images a pathological focal uptake was detected at lymph nodal level in 4/19 patients with no evidence of bone metastases in any patient. The detailed description of sites of lymph nodal and bone ^68^Ga-PSMA and ^68^Ga-DOTA-RM2 uptake are reported in Table 2.

MR images showed intraprostatic disease in all patients, with 10/22 patients also presenting extracapsular extension (ECE) and 7/22 seminal vesicle invasion (SVI). Five out of 22 patients had pathologic pelvic lymph nodes and 1/22 had bone lesions (Table 2). 

Mean ADCmean value (over the patients) of the primary tumours was 0.84 × 10^−3^ mm^2^/s (range: 0.65–1.1), while mean ADCmin value was 0.54 (range: 0.2–0.78) and mean lesion volume was 4.32 cm^3^ (range: 0.49–30.66; Table 5).

### 3.3. Comparisons between ^68^Ga-PSMA PET, ^68^Ga-DOTA-RM2 PET and MRI and Validation with Histology

**Figure 2:**
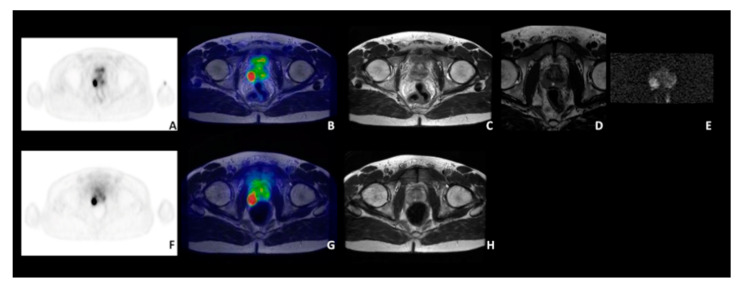
A 53 years-old patient with biopsy-proven PCa (pt n. 10), Gleason score 9 (5 + 4) with a PSA level at diagnosis of 3.13 ng/mL. Concordant ^68^Ga-PSMA PET/MRI (top panel; (**A**): transaxial ^68^Ga-PSMA PET; (**B**): ^68^Ga-PSMA PET/MRI; (**C**): Axial T2-weighted sequence; (**D**): Axial T2-weighted small FOV; (**E**): DWI (b = 1400)) and ^68^Ga-DOTA-RM2 PET/MRI (bottom panel; (**F**): transaxial ^68^Ga-DOTA-RM2 PET; (**G**): ^68^Ga-DOTA-RM2 PET/MRI; (**H**): axial T2-weighted sequence).

Regarding intraprostatic disease, in 16/19 patients the site of the primary prostatic lesion was concordant among the three imaging modalities (see as an example, patient n. 10, Figure 2).

Histological examination was available for 11 of these patients, and, whenever present, confirmed these findings. In the three patients who did not undergo ^68^Ga-DOTA-RM2, ^68^Ga-PSMA and MRI were concordant in identifying the intraprostatic pathological findings (n. 19, n. 20, n. 21, Table 2). 

Among the patients for whom discordant imaging findings were observed, in 1/19 MRI and ^68^Ga-DOTA-RM2 detected bilateral pathological findings, with ^68^Ga-PSMA showing radiotracer uptake only in correspondence of the left lobe (patient n. 16, Table 2). In 1/19 patient (n. 18, Table 2) MRI identified two pathological findings in the right and left side of the prostate, respectively, showing ^68^Ga-PSMA uptake in correspondence of the right lobe and a negative ^68^Ga-DOTA-RM2 PET. These patients have not undergone radical prostatectomy yet, therefore histological examination was not yet available to validate these findings.

Finally, in 1/19, (n. 15, Table 2), ^68^Ga-DOTA-RM2 PET and MRI were concordant in identifying a pathological finding in the right side of the prostate, while ^68G^a-PSMA PET showed a focal uptake in the left lobe; histological examination demonstrated a bilateral prostate cancer with the dominant neoplastic nodule being located in the right lobe.

In terms of the local extension, SVI was detected by MRI in seven patients (n. 3, n. 5, n. 16, n. 18, n. 20, n. 21 and n. 22, Table 2), by ^68^Ga-PSMA in two patients (n. 3, n. 20, Table 2), while no uptake was present on ^68^Ga-DOTA-RM2 images. No histological examination was available for these patients to confirm the imaging findings.

MRI identified ECE in 10 patients (n. 3, n. 5, n. 6, n. 8, n. 10, n. 11, n. 12, n. 14, n. 16, n. 18, Table 2); among the 4/10 patients with the availability of histological confirmation, ECE was confirmed in only 2/4 patients (n. 8, n. 12, Table 2). Both ^68^Ga-PSMA and ^68^Ga-DOTA-RM2 PET are not suitable to identify ECE of PCa, because of the limited spatial resolution compared to MRI.

**Figure 3:**
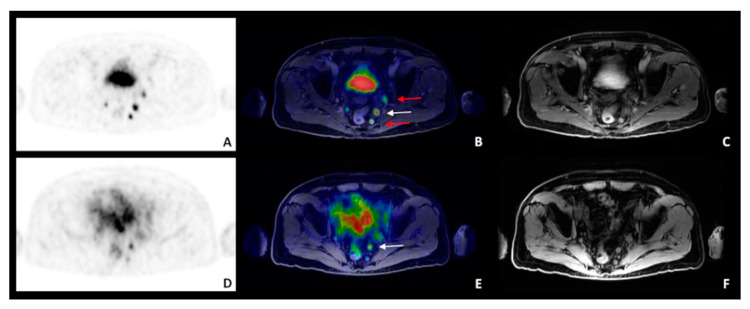
A 75 years-old patient with biopsy-proven PCa (patient n. 3), Gleason score 9 (4 + 5) with a PSA level at diagnosis of 4.73 ng/mL. ^68^Ga-PSMA and ^68^Ga-DOTA-RM2 PET/MRI were discordant in detecting lymphnodal metastases. ^68^Ga-PSMA PET/MRI (top panel; (**A**): transaxial ^68^Ga-PSMA PET; (**B**): ^68^Ga-PSMA PET/MRI; (**C**): post-contrast Water Lava-Flex sequence) showed bilateral pararectal and left external iliac lymphnodal uptake; ^68^Ga-DOTA-RM2 PET/MRI (bottom panel; (**D**): transaxial ^68^Ga-DOTA-RM2 PET; (**E**): ^68^Ga-DOTA-RM2 PET/MRI; (**F**): Water-Lava Flex sequence) showed left pararectal and left external iliac lymphnodal uptake. White arrow indicates the lymph node clearly detected by both tracers; red arrows lymph nodes detected by ^68^Ga-PSMA PET/MRI only.

In terms of lymph nodal involvement, ^68^Ga-PSMA PET resulted positive at lymph nodal level in 7/22 patients (n. 1, n. 3, n. 5, n. 6, n. 7, n. 17, n. 18, Table 2; 26 lesions), while ^68G^a-DOTA-RM2 in 4/19 patients (n. 3, n. 4, n. 5, n. 18, Table 2; 6 lesions) and MRI in 5/22 patients (n. 1, n. 3, n. 5, n. 7, n. 18, Table 2; 8 lesions) (Figure 3, pt n. 3).

**Figure 4:**
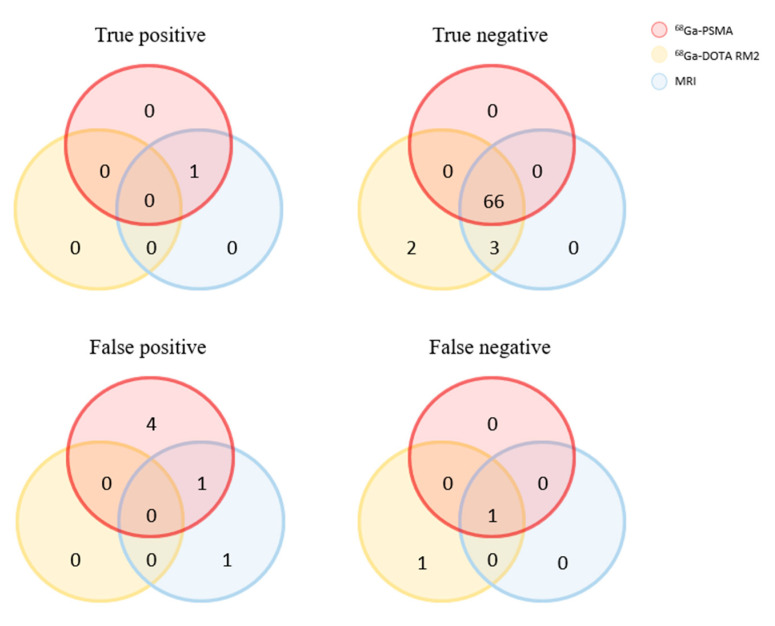
Venn diagram showing the true-positive, false-positive, true-negative and false-negative findings regarding lymph node involvement for all the investigated imaging modalities using histopathological specimens acquired during radical prostatectomy as ground truth.

Histological validation was available for patients n. 1, n. 7 and n. 17, confirming the presence of left external iliac nodal metastases in patient n. 1 and absence of nodal metastases in patients n. 7 and n. 17. Moreover, the lymphnodal involvement described on ^68^Ga-PSMA PET in correspondence of the perivesical fat for patient n. 1 was not confirmed as these lymphnodes were not removed during surgery. Venn diagrams showing the true positive, false positive, true negative and false-negative findings for lymph nodes involvement validated by means of histological specimens for all investigated imaging modalities are depicted in Figure 4.

**Figure 5:**
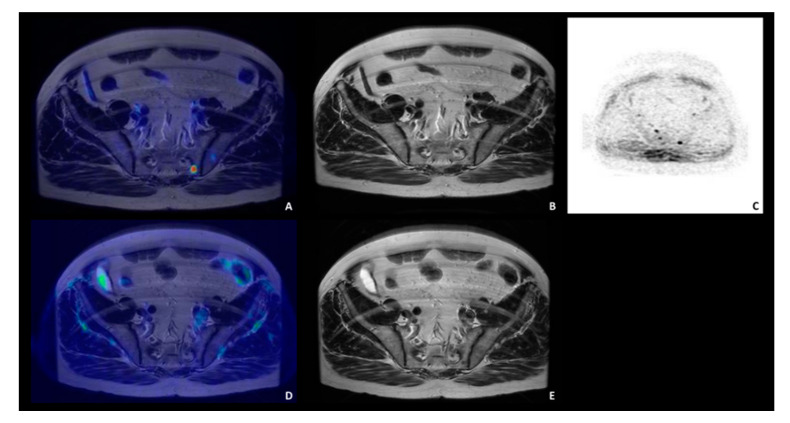
A 59 years-old patient with biopsy-proven PCa (patient n. 6), Gleason score 9 (4 + 5) with a PSA level at diagnosis of 11.0 ng/mL. ^68^Ga-PSMA PET/MRI (top panel; (**A**): ^68^Ga-PSMA PET/MRI; (**B**): axial T2-weighted sequence of the pelvis; (**C**): axial DWI (b = 1000) displayed with inverted greyscale map) showed increased uptake in correspondence of the left sacral ala, where MRI detected a bone metastasis; ^68^Ga-DOTA-RM2 PET/MRI (bottom panel; (**D**): ^68^Ga-DOTA-RM2 PET/MRI; (**E**): axial T2-weighted sequence of the pelvis) did not show any ^68^Ga-DOTA-RM2 in correspondence of the bone metastases.

Regarding distant metastases, ^68^Ga-PSMA showed increased pathological uptake at a bone level in three patients (n. 2, n. 6, n. 18, Table 2), ^68^Ga-DOTA-RM2 did not detect any pathological uptake at bone level. (Figure 5).

**Figure 6:**
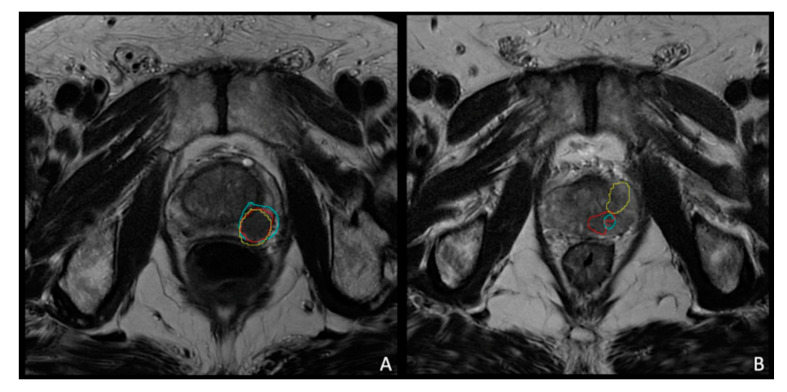
Images representing concordant (**A**) and discordant (**B**) contouring on DICE analysis. (**A**): A 74 years-old patient with biopsy-proven PCa (pt n. 9), Gleason score 9 (5 + 4) with a PSA level at diagnosis of 6.37 ng/mL presenting a prostatic lesion located in the left lobe of the gland. The image shows a concordant identification of the lesion on ^68^Ga-PSMA PET images (blue), ^68^Ga-DOTA-RM2 PET images (yellow) and MRI (red). DICE SCORE: ^68^Ga-PSMA vs. ^68^Ga-DOTA-RM2 = 0.6019, ^68^Ga-PSMA vs. MRI = 0.7581. ^68^Ga-DOTA-RM2 vs. MRI = 0.7220. (**B**): A 52 years-old patient with biopsy-proven PCa (pt n. 14), Gleason score 8 (4 + 4) with a PSA level at diagnosis of 8.04 ng/mL presenting a focal left prostatic. DICE SCORE: ^68^Ga-PSMA vs. ^68^Ga-DOTA-RM2 = 0.2918, ^68^Ga-PSMA vs. MRI = 0.6162. ^68^Ga-DOTA-RM2 vs. MRI = 0.2216.

DICE score was computed to quantitatively assess the overlap between the volume of the primary intra-prostatic lesions manually segmented on ^68^Ga-PSMA PET, ^68^Ga-DOTA-RM2 PET and MR images at the individual level. On average, the DICE score between ^68^Ga-PSMA and MRI = 0.51(range: 0.00–0.79); between ^68^Ga-PSMA and ^68^Ga-DOTA-RM2 = 0.41 (range: 0.05–0.72); while the DICE score between ^68^Ga-DOTA-RM2 and MRI = 0.36 (range: 0.07–0.72). DICE scores for each patient across the investigated modalities are reported in Table 6 (Figure 6).

### 3.4. Correlations between PET Semi-Quantitative and MRI Quantitative Imaging Parameters

None of the investigated semi-quantitative ^68^Ga-PSMA PET parameters significantly correlated with its correspondent parameter on ^68^Ga-DOTA-RM2 PET images, however, the volume of the primary tumour manually segmented on ^68^Ga-PSMA PET was highly correlated with the one manually contoured on MR images (rho = 0.697, *p* = 0.003). MRI quantitative parameters did not correlate with ^68^Ga-DOTA-RM2 PET semi-quantitative parameters.

Tumour volume manually segmented on MR images presented a moderate association with GS that approached the level of significance (rho = 0.419, *p* = 0.053), while ^68^Ga-PSMA PET and ^68^Ga-DOTA-RM2 semi-quantitative parameters did not correlate with any of the considered clinical data (*p*-value ≥ 0.05).

## 4. Discussion

The present pilot study reports our preliminary experience on the use of ^68^Ga-PSMA and ^68^Ga-DOTA-RM2 PET/MRI imaging in high-risk prostate cancer staging.

Few studies have investigated prostate cancer by using both ^68^Ga-PSMA and ^68^Ga-DOTA-RM2 PET so far, both in the staging [15] and restaging setting of the disease [18,19].

In our cohort of patients, differently from all the other published papers, all subjects were studied by using a hybrid PET/MRI scanner both for ^68G^a-PSMA and ^68^Ga-DOTA-RM2 radiotracers [15,18,19].

In fact, among the few published studies that investigated the role of this peculiar multitracer approach in PCa, PET/MRI and PET/CT were used alternatively for ^68^Ga-PSMA and ^68^Ga-DOTA-RM2 PET scans [18,19] or PET/CT were adopted as the only hybrid imaging modality [15].

In the setting of PCa staging, Schollhammer and colleagues reported a clinical case of a patient undergoing PET/CT scans with ^68^Ga-PSMA, ^68^Ga-RM2 and 18F-Choline, while Fassbender et al. used ^68^Ga-PSMA PET/CT and ^68^Ga-Ga-RM2 PET/MRI to study eight patients with a primary diagnosis of PCa [15,24]. The same heterogeneity in terms of the type of scanners used for patients’ scanning can be also observed in the few studies assessing the role of ^68^Ga-PSMA and ^68^Ga-DOTA-RM2 in patients with recurrent PCa. The first study performing a comparative evaluation between these two radiotracers in recurrent PCa is the one by Minamimoto et al. In this pioneering work, comparing the biodistribution of ^68^Ga-PSMA-11 and ^68^Ga-RM2 in a small cohort of patients with biochemically recurrent PCa, PET/CT was adopted for ^68^Ga-PSMA studies while PET/MRI scanner was used for ^68^Ga-DOTA-RM2 PET acquisitions [18].

Similarly, Baratto et al. recently published a study on the use of ^68^Ga-PSMA and ^68^Ga-DOTA-RM2 in a cohort of patients with recurrent PCa and compared the diagnostic performances of these two radiotracers. They showed that ^68^Ga-PSMA11 and 18F- DCFPyL might have a complementary role as they detect different sites of disease recurrence. Notably, the group used a PET/MR scanner only for ^68^Ga-RM2 imaging and regarding PSMA PET/CT scans, ^68^Ga-PSMA11 or 18F- DCFPyL were alternatively used [19].

The use of a PET/MRI scanner in the staging phase of PCa allows to perform a diagnostic MRI on the pelvic region, thus obtaining all the necessary morphological and multiparametric information for accurate identification and characterisation of the primary tumour. Moreover, the possibility to simultaneously acquire a PET scan with two different radiotracers assessing different metabolic pathways provides additional information regarding primary tumour characteristics, together with a whole-body evaluation of the disease. Finally, the use of a PET/MRI scanner rather than PET/CT scanner strongly reduces the radiation exposure for the patient [25].

Differently from other groups that investigated the dual tracer approach of ^68^Ga-PSMA and ^68^Ga-DOTA-RM2 in PCa staging, or restaging, using a PET/CT scanner [15,18,19], one of the most relevant patients’ advantages in the present study relies on the possibility to have received a diagnostic MRI simultaneously acquired to the PET image acquisition. In fact, MRI is expected to increase the diagnostic accuracy of PET imaging for local staging (ECE and SVI) [26], and the information derived from both modalities could be incorporated into clinical nomograms to significantly enhance the pre-operative staging accuracy [27,28]. Moreover, MRI shows excellent diagnostic performance in the detection of bone metastases [29]. If used in combination with PET, MRI could provide complementary information on the bone disease when PET findings are equivocal or when metastatic lesions do not show significant PSMA uptake. Finally, WB-MRI could be of added value in monitoring the response to loco-regional or systemic treatments [30,31].

In our cohort of patients, the primary PCa was detected in 18/19 patients by all three imaging modalities. In our cohort of patients, histological examination was available only for a minority of subjects and, regarding the intraprostatic disease, was used as the gold standard to confirm the findings detected by the three imaging modalities. In one patient (n. 15) MRI and ^68^Ga-DOTA-RM2 identified a lesion in the right prostatic lobe, with ^68^Ga-PSMA being positive in the left lobe. Histological examination reported bilateral prostatic neoplasia with a right dominant nodule. The discrepancies observed between ^68^Ga-PSMA and ^68^Ga-RM2 might reflect a complementary role of these imaging modalities in identifying different intraprostatic foci of disease presenting different metabolic patterns, thus enlightening a synergic role of ^68^Ga-PSMA and ^68^Ga-DOTA-RM2 in prostate cancer, in line with previously published data [15,18,19].

For instance, Fassbender et al. in their cohort of eight patients with primary PCa undergoing ^68G^a-PSMA PET/CT and ^68^Ga-Ga-RM2 PET/MRI concluded that the qualitative findings of PET scans could provide combined relevant information. In their study, both radiotracers partially showed the same tumour region and, in some cases, different tumour parts, thus providing a better PCa characterisation and reflecting the heterogeneous and sometimes polyclonal behaviour that characterise PCa [15]. Similarly, the results reported by Baratto and colleagues, comparing RM2-PET and PSMA-PET in patients with biochemically recurrent PCa and by Iagaru and colleagues in patients with newly diagnosed intermediate- or high-risk prostate cancer suggested that the use of both ^68^Ga-PSMA and ^68^Ga-DOTA-RM2 provided different and complementary information on PCa [19,32].

To investigate the correspondence of the intra-prostatic findings referable to the site of the primary tumour across modalities, DICE score between manually segmented primary intra-prostatic tumour volumes on ^68^Ga-PSMA, ^68^Ga-DOTA-RM2 PET and MR images were calculated. Volumes of the primary tumours, as defined in all the investigated imaging modalities largely overlap. The highest mean DICE score was the one between ^68^Ga-PSMA PET and MRI. This may be partially explained by the fact that ^68^Ga-DOTA-RM2 tumour volumes were generally smaller, and partially by the fact that ^68^Ga-PSMA PET and MRI were simultaneously acquired, thus being intrinsically co-registered. Conversely, an automatic co-registration tool on 3D Slicer, with manual adjustments when needed, was used to overlap ^68^Ga-DOTA-RM2 PET images to ^68^Ga-PSMA and MR diagnostic images. A residual component of noise might have hampered the computation of the DICE score, therefore resulting in a minor overlap between ^68^Ga-DOTA-RM2 PET and the other images.

Spearman correlations between multitracer PET and MRI parameters revealed a significant, strong, correlation between tumour volume manually segmented on ^68^Ga-PSMA PET and MR images. No significant associations between parameters derived from PET with different radiotracers were found. This is in contrast with the strong association between ^68^Ga-PSMA PET and ^68^Ga-DOTA-RM2 PET SUVmax and SUVmean reported by Minamimoto et al. in 2016 [18]. However, that pioneering work relied on a very small sample (*n* = 7) of patients presenting with biochemical recurrence. Future studies with larger cohorts of patients are needed to unravel the possible association between semi-quantitative parameters derived from ^68^Ga-PSMA and ^68^Ga-DOTA-RM2 PET. Furthermore, concerning the correlation between imaging parameters and clinical data, a moderate correlation between tumour volume manually segmented on MR images and GS at biopsy that approached the level of significance (*p* = 0.053) was detected. All the other tested correlations resulted non-significant and this is in line with what is reported by Fassbender et al. [15]. These results have to be interpreted with great caution and more evidence is needed before speculating on the clinical utility of these findings since our sample was small and quantitative analyses might be susceptible to lack of statistical power. Future studies, with larger samples, will allow for the unraveling of the possible association between semi-quantitative PET, quantitative MRI parameters and clinical data.

Some limitations should be pointed out regarding the present study. First of all, histopathological correlation with the post-surgical specimen was available only for those patients who have undergone radical prostatectomy. Therefore, being the present analysis a pilot study with only a preliminary evaluation of collected imaging and histological data, the analysis will certainly be improved as soon as all histological data are available, with also a detailed co-registration between imaging and histopathological data as well as correlations between imaging findings and histological examinations. Another limitation of this study is the low number of patients. However, besides the fact that the few papers already published on PCa staging and using both ^68^Ga-PSMA and ^68^Ga-RM2 PET radiotracers included a number of patients even lower than the one presented in the present paper [15], we consider that these preliminary data are interesting to underlie the potential complementary and synergic role of the two different PET radiotracers together with mp-MRI.

## 5. Conclusions

Based on the results of the present study, a potential complementary role of ^68^Ga-PSMA and ^68^Ga-DOTA-RM2 in PCa staging can be enlightened, in view of the different findings detected by the two imaging modalities in some of the patients included in our cohort. In fact, the possibility to identify different sites of disease by using a multitracer approach certainly improves the disease characterization and therefore it may ultimately have an impact on patients’ management and follow-up. These findings should be validated on larger cohorts of patients to definitively assess the utility of hybrid ^68^Ga-PSMA PET/MRI and ^68^Ga-DOTA-RM2 PET/MRI in the clinical management of PCa.

## Figures and Tables

**Table 1:** Patients’ characteristics.

n.	Age	PSA Level at Diagnosis (ng/mL)	GS on Biopsy	ISUP Grade on Biopsy	Clinical T Stage
1	71	5.04	7 (4 + 3)	3	T2c
2	80	11.13	8 (3 + 5)	4	T1
3	74	4.73	9 (4 + 5)	5	T2a
4	71	5.80	7 (4 + 3)	3	T2c
5	69	3.03	9 (5 + 4)	5	T1
6	59	11.00	9 (4 + 5)	5	T3b
7	75	5.33	8 (4 + 4)	5	T2a
8	62	3.85	8 (4 + 4)	4	T1
9	74	6.37	9 (5 + 4)	5	T2a
10	53	3.13	9 (4 + 5)	5	T2b
11	69	5.31	9 (5 + 4)	5	T2c
12	74	5.03	8 (4 + 4)	4	T2a
13	64	4.40	8 (4 + 4)	4	T1
14	52	8.04	8 (4 + 4)	4	T2a
15	66	6.37	9 (4 + 5)	5	T2a
16	66	2.43	9 (4 + 5)	5	T3b
17	55	2.69	9 (5 + 4)	5	T2c
18	55	5.24	9 (4 + 5)	5	T2c
19	76	8.19	9 (4 + 5)	5	T2a
20	54	26.17	8 (4 + 4)	4	T1
21	60	16.34	9 (4 + 5)	5	T2a
22	57	9.49	8 (4 + 4)	4	T1

PSA: Prostate Specific Antigen; GS: Gleason Score; ISUP: International Society of Urological Pathology.

**Table 2:** TNM findings of ^68^Ga-PSMA, ^68^Ga-RM PET/MRI and histological validation.

n.	Histological Specimen			^68^Ga-PSMA			^68^Ga-DOTA-RM2			MRI		
	T	N	M	T	N	M	T	N	M	T	N	M
1	Prostate (bilateral), ECE, left SVI	Left external iliac LN	NA	Prostate (bilateral)	Left external iliac, left Perivescical fat	Negative	Prostate (bilateral)	Negative	Negative	Prostate (bilateral)	Left external iliac	Negative
2	Prostate (bilateral), ECE	Negative	NA	Prostate (bilateral)	Negative	Right iliac bone	Prostate (bilateral)	Negative	Negative	Prostate (bilateral)	Negative	Negative
3	NA	NA	NA	Prostate (multiple bilateral focal uptake), SVI	Left external iliac, bilateral perirectal, presacral	Negative	Prostate (bilateral)	left iliac, left perirectal	Negative	Prostate (bilateral), SVI, ECE	Left external iliac, left pararectal,	Negative
4	Prostate (bilateral, right dominant nodule)	Negative	NA	Prostate (right)	Negative	Negative	Prostate (right)	hilomediastinic	Negative	Prostate (right, multiple foci)	Negative	Negative
5	NA	NA	NA	Prostate (bilateral)	Left perivescical, bilateral obturator, left external iliac	Negative	Prostate (bilateral)	Left external iliac, left obturator	Negative	Prostate (bilateral), SVI, ECE	left obturator, right obturator, external bilateral iliac	Negative
6	NA	NA	NA	Prostate (left)	Left perirectal	Right ribs, left sacral ala	Prostate (left)	Negative	Negative	Prostate (left), ECE	Negative	Right ribs, left sacral ala
7	Prostate (right)	Negative	NA	Prostate (right)	Bilateral external iliac, right common iliac	Negative	Prostate (right)	Negative	Negative	Prostate (right)	Bilateral iliac	Negative
8	Prostate (bilateral, right dominant nodule), ECE	Negative	NA	Prostate (right)	Negative	Negative	Prostate (right)	Negative	Negative	Prostate (right), ECE	Negative	Negative
9	Prostate (bilateral, left dominant nodule)	Negative	NA	Prostate (left)	Negative	Negative	Prostate (left)	Negative	Negative	Prostate (left)	Negative	Negative
10	Prostate (right)	Negative	NA	Prostate (right)	Negative	Negative	Prostate (right)	Negative	Negative	Prostate (right), ECE	Negative	Negative
11	Prostate (bilateral, left dominant nodule)	Negative	NA	Prostate (left)	Negative	Negative	Prostate (left)	Negative	Negative	Prostate (left), ECE	Negative	Negative
12	Prostate (bilateral, left dominant nodule), ECE	Negative	NA	Prostate (left)	Negative	Negative	Prostate (left)	Negative	Negative	Prostate (left), ECE	Negative	Negative
13	Prostate (bilateral, right dominant nodule), ECE, SVI	Left common iliac LN	NA	Prostate (right)	Negative	Negative	Prostate (right)	Negative	Negative	Prostate (right, bifocal)	Negative	negative
14	NA	NA	NA	Prostate (left)	Negative	Negative	Prostate (left)	Negative	Negative	Prostate (left), ECE	Negative	Negative
15	Prostate (bilateral, right dominant nodule), ECE	Negative	NA	Prostate (left)	Negative	Negative	Prostate (right)	Negative	Negative	Prostate (right)	Negative	Negative
16	NA	NA	NA	Prostate (left)	Negative	Negative	Prostate (right and left)	Negative	Negative	Prostate (right and left), ECE, SVI	Negative	Negative
17	Prostate (bilateral, right dominant nodule), ECE	Negative	NA	Prostate (right)	External iliac	Negative	Prostate (right)	Negative	Negative	Prostate (right)	Negative	Negative
18	NA	NA	NA	Prostate (right)	Left supraclavicular, subcarinal, lomboaortic, paracaval, interaortocaval, bilateral iliac, mesorectal	C3, right iliac ala, left posterior iliac crest	Negative	Left retroclavear, lomboaortic, bilateral iliac	Negative	Prostate (left and right), ECE, SVI	Pelvic, left external iliac	Negative
19	NA	NA	NA	Prostate (apex left paramedian)	Negative	Negative	NA	NA	NA	Prostate (apex left paramedian)	Negative	Negative
20	NA	NA	NA	Prostate (right and left), SVI	Negative	Negative	NA	NA	NA	Prostate (right and left), SVI	Negative	Negative
21	NA	NA	NA	Prostate (right)	Negative	Negative	NA	NA	NA	Prostate (right), SVI	Negative	Negative
22	NA	NA	NA	Prostate (multiple bilateral focal uptake)	Negative	Negative	Prostate (multiple bilateral focal uptake)	Negative	Negative	Prostate (bilateral), SVI	Negative	Negative

LN: Lymph Node; ECE: extracapsular extension; SVI: Seminal Vesicles Invasion; NA: not available (for histological specimen meaning that either the patient did not perform radical prostatectomy or no specimens were removed from that specific region).

**Table 3:** High statistic ^68^Ga-PSMA PET parameters.

n.	SUV Max	SUV Mean40	SUV Mean50	SUV Mean60	MTV 40 (cm^3^)	MTV 50 (cm^3^)	MTV 60 (cm^3^)	Volume (cm^3^)
1	16.71	9.08	10.36	13.67	1.74	0.842	0.163	4.83
2	37.04	23.12	25.31	28.96	0.19	0.136	0.081	3.95
3	20.19	14.55	14.86	16.04	1.95	1.85	1.38	31.16
4	4.08	3.02	3.04	3.18	3.39	3.34	2.85	1.61
5	43.44	25.92	28.01	30.76	0.95	0.706	0.434	6.49
6	35.32	21.87	24.21	25.93	5.29	3.83	2.77	10.19
7	12.97	7.22	8.04	8.92	1.57	1.03	0.57	4.91
8	21.78	13.39	14.65	17	1.06	0.787	0.434	2.69
9	29.47	17.58	20.14	22.82	1.33	0.842	0.516	3.82
10	19.38	11.35	13.03	14.17	1.85	1.11	0.76	5.58
11	16.23	9.82	10.62	11.97	2.36	1.76	1	3.62
12	40.08	24.4	27.53	29.8	0.597	0.407	0.299	2.60
13	21.26	14.13	14.83	16.22	0.407	0.353	0.244	1.33
14	8.64	5.1	5.77	6.32	2.33	1.49	0.977	1.19
15	7.18	4.03	4.57	5.11	2.47	1.47	0.814	1.98
16	4.75	3.31	3.38	3.59	2.04	1.93	1.52	0.78
17	14.52	8.52	9.62	11.26	1.68	1.11	0.543	2.12
18	8.41	4.8	5.42	5.94	15.53	9.77	5.97	7.65
19	40.93	24.88	28.28	32.31	0.652	0.434	0.271	3.73
20	60.97	35.58	40.58	44.81	4.1	2.55	1.57	8.54
21	73.92	43.85	50.37	54.61	1.06	0.679	0.462	4.20
22	38.15	21.82	24.99	28.1	3.53	2.12	1.22	11.55
Mean	26.16	15.79	17.62	19.61	2.55	1.75	1.13	5.66
Range	4.08–73.92	3.02–43.85	3.04–50.37	3.18–54.61	0.19–15.53	0.136–9.77	0.081–5.97	0.78–31.16

SUV: Standardised Uptake Value; MTV: Metabolic Tumour Volume; Volume: manually segmented volume on 3D Slicer.

**Table 4:** High statistic ^68^Ga-DOTA-RM2 PET parameters.

n.	SUV Max	SUV Mean40	SUV Mean50	SUV Mean60	MTV 40 (cm^3^)	MTV 50 (cm^3^)	MTV 60 (cm^3^)	Volume (cm^3^)
1	21.2	12.63	14.29	15.41	2.09	1.38	0.977	5.47
2	15.91	8.86	10.18	11.9	1.52	0.869	0.407	2.00
3	13.75	7.47	8.78	10.12	1.19	0.624	0.326	2.05
4	12.48	7.09	8.36	8.97	4.02	2.25	1.6	4.45
5	24.3	15.88	17.59	18.51	5.24	3.96	3.26	4.57
6	11.66	7.92	8.64	9.08	7.09	5.62	4.7	3.35
7	24.55	15.51	17.67	18.8	0.787	0.543	0.434	4.48
8	22.94	17.13	17.44	17.96	1.22	1.17	1.06	1.36
9	9.91	5.45	6.61	7.31	4.34	2.06	1.33	1.94
10	30.93	20.33	22.62	23.55	1.9	1.38	1.17	7.43
11	17.68	10.01	11.79	13.39	4.89	2.71	1.52	6.69
12	4.22	2.88	2.99	3.09	4.86	4.29	3.75	2.21
13	12.97	7.58	8.71	9.4	0.597	0.353	0.244	1.75
14	5.74	4.62	4.63	4.69	4.75	4.72	4.53	1.59
15	10.67	6.8	7.08	7.7	4.67	4.05	2.63	3.38
16	16.53	10.98	12.33	13.3	1.79	1.3	1	0.58
17	10	7.8	7.8	7.85	2.04	2.04	1.98	1.61
18	NA	NA	NA	NA	NA	NA	NA	NA
19	NA	NA	NA	NA	NA	NA	NA	NA
20	NA	NA	NA	NA	NA	NA	NA	NA
21	NA	NA	NA	NA	NA	NA	NA	NA
22	23.7	14.56	15.86	17.31	3.15	2.39	1.57	3.69
Mean	15.40	9.86	10.90	11.69	3.13	2.37	1.88	3.26
Range	3.39–30.93	2.88–20.33	2.99–22.62	3.09–23.35	0.60–7.09	0.35–5.62	0.24–4.70	0.58–7.43

SUV: Standardised Uptake Value; MTV: Metabolic Tumour Volume; Volume: Manually segmented volume on 3D Slicer.

**Table 5:** MRI quantitative parameters.

n.	ADC Min (10^−3^ mm^2^/s)	ADC Mean (10^−3^ mm^2^/s)	Volume (cm^3^)
1	0.4	0.8	3.36
2	0.4	0.78	1.80
3	0.49	0.86	30.66
4	0.5	1.1	0.51
5	0.44	0.82	7.95
6	0.33	0.78	7.78
7	0.5	0.65	1.12
8	0.56	0.78	0.80
9	0.2	0.69	2.50
10	0.61	0.83	3.38
11	0.61	0.99	3.31
12	0.68	0.95	1.15
13	0.34	0.66	0.49
14	0.5	0.85	1.86
15	0.67	1	1.01
16	0.64	0.80	6.94
17	0.74	0.82	0.98
18	0.61	0.83	7.53
19	0.78	0.90	1.16
20	0.48	0.82	4.38
21	0.72	0.83	1.92
22	0.73	0.86	4.47
Mean	0.54	0.84	4.32
Range	0.2–0.78	0.65–1.1	0.49–30.66

ADC: Apparent Diffusion Coefficient; Volume: Manually segmented volume on 3D Slicer.

**Table 6:** DICE scores.

n.	^68^Ga-PSMA vs. MRI	^68^Ga-DOTA-RM2 vs. MRI	^68^Ga-PSMA vs. ^68^Ga-DOTA-RM2
1	0.7151	0.5189	0.6521
2	LNI	0.6052	LNI
3	0.7684	0.0859	0.1188
4	0.0000	0.0728	0.1524
5	0.7354	0.3723	0.4544
6	0.7907	0.5872	0.4856
7	0.3697	0.3529	0.5571
8	0.4178	0.4331	0.5981
9	0.7581	0.7220	0.6019
10	0.7057	0.5761	0.7174
11	0.7810	0.6259	0.6828
12	0.6056	0.4023	0.5357
13	0.5013	0.2749	0.3654
14	0.6162	0.2216	0.2918
15	LNI	LNI	0.2157
16	0	0.0971	0.0514
17	0.6062	0.1040	0.1461
18	0.0671	LNI	LNI
19	0.4751	NA	NA
20	0.5526	NA	NA
21	0.5769	NA	NA
22	0.0997	0.3667	0.3667
Mean	0.5071	0.3560	0.4114
SD	0.2677	0.2292	0.2292

LNI: Lesion not identified in a specific modality, DICE score could not be calculated, NA: Not available.
